# Association between oxidative balance score and prevalence rates of thyroid dysfunction and autoimmune thyroiditis among U.S. adults: evidence from epidemiological studies

**DOI:** 10.3389/fnut.2025.1592577

**Published:** 2025-05-15

**Authors:** Jie Wu, Xuqin Dong, Qingkai Yang, Junxian Niu, Chuyu Jia, Xin Li, Zhuyan Li

**Affiliations:** ^1^Department of Thyroid Surgery, Shanxi Provincial People's Hospital Affiliated to Shanxi Medical University, Taiyuan, China; ^2^Department of Physical Examination Center, Shanxi Provincial People's Hospital Affiliated to Shanxi Medical University, Taiyuan, China

**Keywords:** oxidative balance score, thyroid dysfunction, oxidative stress, subclinical hyperthyroidism, antioxidant

## Abstract

**Background:**

Oxidative stress can impact the synthesis, metabolism, and signaling pathways of thyroid hormones. The Oxidative balance score (OBS) is an indicator used to measure an individual’s oxidative stress status by assessing the levels of oxidative and antioxidant substances in diet and lifestyle factors. This study aimed to explore the relationship between OBS and the prevalence of thyroid dysfunction.

**Methods:**

This study investigated the relationship between OBS and the prevalence of thyroid dysfunction using data from 6,268 participants in the 2007–2012 National Health and Nutrition Examination Survey (NHANES). Weighted multivariate logistic regression and restricted cubic spline (RCS) regression were employed to analyze this association, assessing both linear and potential non-linear relationships.

**Results:**

After adjusting for covariates, our research revealed that the prevalence of subclinical hyperthyroidism (SCHyper) decreased by 7.4% for each additional OBS unit[OR = 0.926, 95% CI = (0.884, 0.971), *p* = 0.002]. When the OBS was categorized, we also discovered that individuals with the highest OBS exhibited a 62.3% reduced risk of developing SCHyper in comparison to those with the lowest OBS. Similarly to OBS, the prevalence of SCHyper diminished by 7.7% for every extra unit of OBS added to the diet [OR = 0.923, 95% CI = (0.874, 0.974), *p* = 0.005]. A stratified analysis revealed that the protective effect of OBS against SCHyper was particularly strong in women, non-Hispanic White people, individuals with poverty to income ratio (PIR) greater than 3.5, and those with a Urinary iodine concentration (UIC) below 300 ug/L (all *P* for interaction < 0.05). Additionally, our investigation revealed a U-shaped curve relationship between OBS and SCHyper.

**Conclusion:**

Research indicates a negative correlation between OBS and the risk of SCHyper, suggesting that a higher intake of antioxidants and reduced exposure to pro-oxidants may help lower the risk of SCHyper. These results offer new insights into the prevention and treatment of patients with SCHyper.

## 1 Introduction

Thyroid hormones are crucial for the growth, brain development, and energy metabolism of the body. Thyroid dysfunction can manifest as hypothyroidism, where the body lacks sufficient thyroid hormones, resulting in symptoms such as fatigue, weight gain, low metabolic rate, and depression (1). On the opposite end of the spectrum is hyperthyroidism, characterized by an overproduction of these hormones, which can cause symptoms like weight loss, anxiety, and increased heart rate (2). Autoimmune thyroiditis is a chronic condition in which the immune system mistakenly attacks the thyroid gland, leading to inflammation and damage (3). The prevalence of thyroid dysfunction and autoimmune thyroid disease varies by factors including sex (4), age (5), lifestyle (6), diet (7, 8), and other factors (9–11).

Oxidative stress (OS) occurs when the production of reactive oxygen species (ROS) and free radicals in the body exceeds the scavenging capacity of the antioxidant defense system, leading to cellular and tissue damage. Mitochondria serve as the primary sites for the production of ROS and are also the primary targets of these potentially harmful molecules, which can induce mitochondrial dysfunction and consequently lead to metabolic disorders. Previous research has indicated that OS is associated with both hyperthyroidism and hypothyroidism (12). By supplementing with antioxidants, adjusting one’s diet, and altering lifestyle habits, it is possible to alleviate the adverse impact of oxidative stress on the thyroid, improving thyroid function and related symptoms (13).

The Oxidative balance score (OBS) is an indicator used to measure an individual’s oxidative stress status by assessing the levels of oxidative and antioxidant substances in diet and lifestyle factors (14). Higher OBS scores indicate higher antioxidant exposure and lower pro-oxidant exposure. Compared to a single marker, it can assess the oxidative state more comprehensively (15). Research has indicated correlations between OBS and the risk of various chronic diseases, such as cardiovascular disease (16), mortality rate in patients with diabetes (17), breast cancer (18), depression (19), kidney stones (20), and female infertility (21). However, it is currently unclear whether there is an association between OBS and thyroid diseases.

Although a recent study has shown a significant inverse correlation between OBS and levels of thyroid hormones, such as TT4 and FT4, among American adults (22), the impact of OBS on thyroid function only in normal individuals. Therefore, this study seeks to investigate the association between OBS and the prevalence of thyroid dysfunction in U.S. adults, utilizing data from the National Health and Nutrition Examination Survey (NHANES). The aim was to provide valuable insights into OBS as a modifiable risk factor for thyroid health, which could inform public health strategies aimed at preventing and managing thyroid disorders.

## 2 Methods

### 2.1 Study population

NHANES is a comprehensive and nationally representative cross-sectional survey that has been meticulously designed to evaluate and assess the health and nutritional status of the entire population residing within the United States. NHANES collects vital data on the prevalence of major diseases and conditions, dietary habits, nutritional trends, and health-related behaviors across the nation through a combination of interviews and physical examinations. Conducted by the National Center for Health Statistics (NCHS) under the Centers for Disease Control and Prevention (CDC), all participants provided informed consent prior to the start of the study.

**Figure 1:**
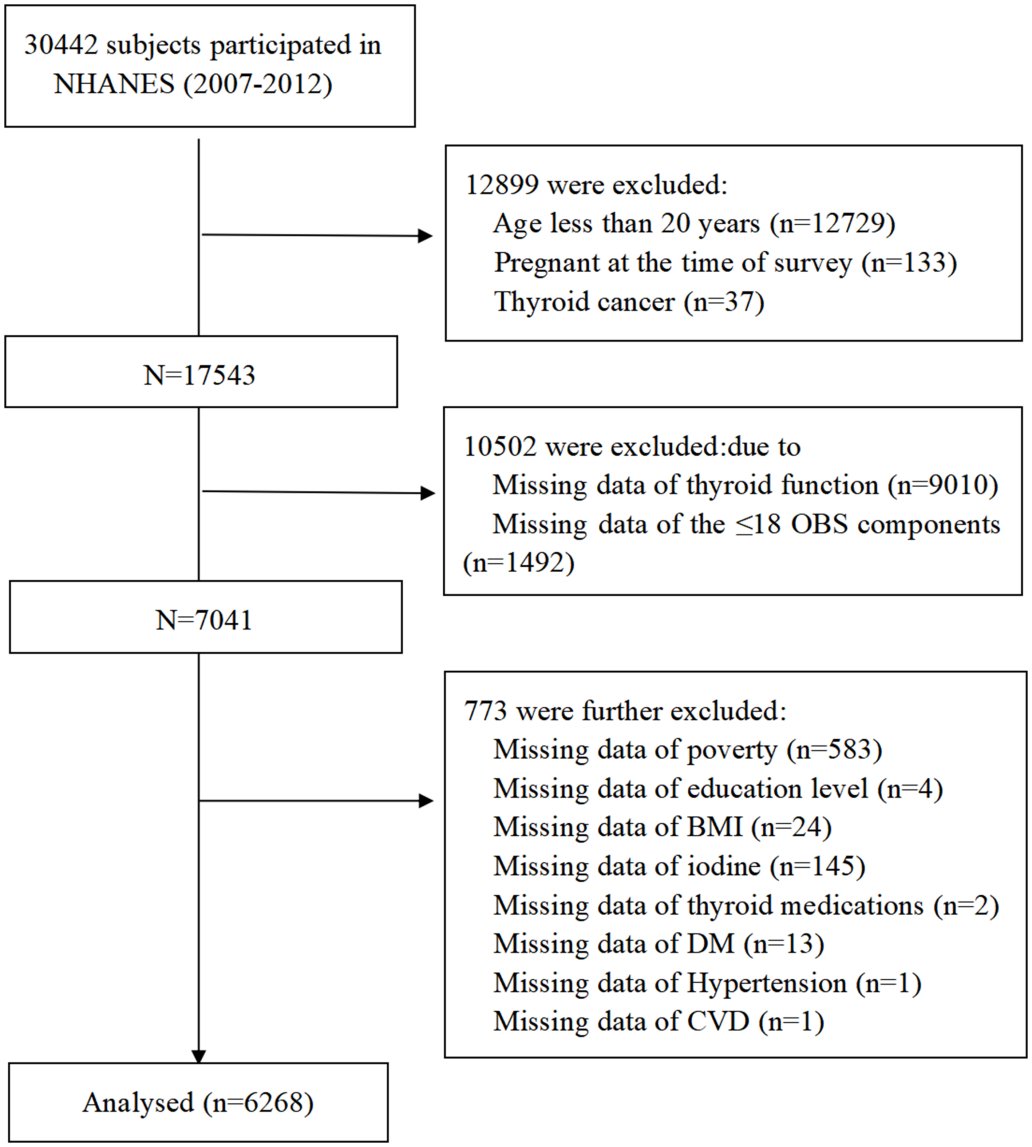
Flowchart of study population.

Data from three cycles (2007–2008, 2009–2010, and 2011–2012), totaling 30,442 participants, were initially considered. This study applied the following exclusion criteria: (1) participants younger than 20 years or currently pregnant; (2) participants with thyroid cancer; (3) participants without available thyroid measurement data; (4) participants with ≤ 18 OBS components (23); (5) participants with missing covariate data. Finally, 6,268 participants were included in the analysis (Figure 1).

### 2.2 Serum thyroid measure and thyroid dysfunction

The assessment of serum thyroid hormones encompasses the evaluation of thyroid stimulating hormone (TSH), free thyroxine (FT4), free triiodothyronine (FT3), total thyroxine (TT4), total triiodothyronine (TT3), thyroid peroxidase antibodies (TPOAb) and thyroglobulin antibodies (TgAb). The thyroid blood specimens analyzed in this study were obtained from the publicly available NHANES database. The NCHS adheres to standardized protocols for sample collection, processing, and biomarker measurement, which are meticulously documented in the NHANES Laboratory Procedures Manual. The reference ranges for TSH, FT4, and FT3 were 0.4–4.5 mIU/L, 9–25 pmol/L, and 2.5–3.9 pg./mL, respectively, according to the previous studies and clinical practice guidelines (24–26). Participants who had not received thyroid hormone replacement therapy or antithyroid drugs and whose TSH value was normal range were classified as having normal thyroid function (2). We defined thyroid dysfunction and autoimmune thyroid disease as follows: Hashimoto’s thyroiditis (HT): participants with TPOAb > 9 IU/mL and TgAb > 115 IU/mL were considered positive (27). Autoimmune thyroiditis (AIT): participants with TPOAb > 9 IU/mL and TgAb > 4 IU/mL were considered positive (28). Subclinical hypothyroidism (SCH): participants with TSH ≥ 4.5 mIU/L and FT4 level within 9–25 pmol/L, but they were not taking any thyroid drugs. Subclinical hyperthyroidism (SCHyper): participants with TSH < 0.4 mIU/L and FT4 level within 9–25 pmol/L and FT3 within 2.5–3.9 pg./mL, but they were not taking any thyroid drugs. Hypothyroidism: (1) participants who self-reported take medication for hypothyroidism (levothyroxine, liothyronine or desiccated thyroid) or (2) participants with TSH ≥ 4.5 mIU/L and FT4 level < 9 pmol/L, but they were not taking any thyroid drugs. Hyperthyroidism: (1) participants who self-reported take antithyroid drugs (propylthiouracil or methimazole) or (2) participants with TSH < 0.4 mIU/L and FT4 level > 25 pmol/L, and did not take any thyroid drugs or (3) participants with TSH < 0.4 mIU/L and FT3 level > 3.9 pmol/L, but they were not taking any thyroid drugs.

### 2.3 Oxidative balance score

The total OBS components was formed from dietary and lifestyle components, which contains 16 dietary factors (vitamin B6, vitamin B12, vitamin C, vitamin E, calcium, iron, zinc, selenium, magnesium, copper, total folate, total fat, niacin, riboflavin, dietary fiber and carotene) and 4 lifestyle components (alcohol consumption, smoking, body mass index (BMI), and physical activity). And further categorized into 15 antioxidants and 5 pro-oxidants. Dietary components and alcohol consumption were calculated based on the average of two 24-h dietary recalls. Cotinine is a useful biomarker for smoking because of its long half - life and its ability to indicate both active and passive smoking exposure. Total physical activity was measured as metabolic equivalent (MET) score and the following formula: MET × frequency of each physical activity per week × duration of each physical activity (29, 30). The total OBS score was determined by adding the scores for each component, with a higher OBS score indicating greater exposure to antioxidants.

### 2.4 Covariates

Building upon previous studies, we considered a range of factors as potential confounders, encompassing demographic, socioeconomic, dietary, and health-related variables. These factors included age, gender, race/ethnicity, education level, poverty to income ratio (PIR), iodine status, diabetes, hypertension, and cardiovascular disease (CVD).

Participants were stratified into three age cohorts: 20–40 years, 41–60 years, and over 60 years. Ethnicity/race was classified as non-Hispanic White, non-Hispanic Black, Mexican American, and other. Educational attainment was classified into three tiers: less than high school, high school graduate, and more than high school. PIR was also grouped into three categories: ≤ 1.3, 1.3 **<** PIR ≤ 3.5, and > 3.5. Urinary iodine concentration (UIC) was used to assess iodine status and was categorized as <100, 100–299 and ≥ 300 ug/L (31). Diagnosed through index measurements, medication use, and self-reporting, hypertension and diabetes, along with CVD identified solely by self-reporting.

### 2.5 Statistical analysis

Due to the complexity of the sampling survey in NHANES, a weighted statistical analysis was applied to each study participant to ensure representativeness. Categorical variables were summarized by frequency and percentage, whereas continuous variables were reported as means with standard deviations (SD). The weighted chi-square test was utilized to evaluate categorical differences, whereas continuous variables were assessed using one-way analysis of variance (ANOVA).

In the multivariable logistic regression analysis examining the relationship between OBS and the prevalence of thyroid dysfunction, Model 1 was not adjusted for any covariates. Model 2 incorporated adjustments for various factors, including gender, race/ethnicity, education level, age, UIC, and PIR. Model 3, the fully adjusted model, was adjusted for the variables in Model 2 and further adjusted to account for additional potential confounders, such as diabetes, hypertension, and cardiovascular disease (CVD). Restricted cubic spline (RCS) regression was employed to analyze both linear and potential non-linear relationships. Subgroup analyses and interaction tests were employed for each covariate to assess the heterogeneity in the relationship between OBS and the prevalence of thyroid dysfunction. A two-sided *p* value of < 0.05 was considered statistically significant. All analyses were conducted using R software (version 4.4.1).

## 3 Results

### 3.1 Baseline participant characteristics

We included 6,268 participants who were 20 years of age or older from the NHANES (2007–2012). Table 1 displayed the baseline characteristics of participants categorized according to OBS quartiles. Participants in different OBS quartiles exhibited significant differences in age, race, PIR, and education level (all *p* < 0.05). As the OBS increased, the incidence of diseases including diabetes, hypertension and CVD gradually decreased. Moreover, participants in the highest OBS quartile showed a significantly lower prevalence of SCHyper compared to those in the lowest OBS quartile (*p* < 0.0001). However, there were no significant differences in the prevalence of HT, AIT, SCH, hyperthyroidism and hypothyroidism among the quartile groups. We also conducted an identical analysis on both dietary and lifestyle OBS. Consistent with previous findings for the total OBS, our results further revealed that dietary OBS was found to be associated with a decreased incidence of SCHyper (Supplementary Tables 1, 2).

**Table 1:** Baseline characteristics of participants categorized according to OBS quartiles.

Characteristics	OBS quartiles					*p*-value
	Overall	Q1	Q2	Q3	Q4	
Age(years)						**0.008**
20–40	2,132(39.227)^a^	532(39.330)	453(36.937)	612(37.814)	535(42.408)	
41–60	2076(38.990)	555(36.744)	439(38.084)	583(38.981)	499(41.693)	
>60	2060(21.784)	673(23.926)	490(24.979)	545(23.206)	352(15.899)	
Gender						0.872
Female	3,167(52.823)	857(52.362)	718(51.756)	895(53.555)	697(53.268)	
Male	3,101(47.177)	903(47.638)	664(48.244)	845(46.445)	689(46.732)	
Race						**< 0.0001**
White	3,089(71.277)	774(66.079)	656(68.636)	876(71.329)	783(77.875)	
Black	1,239(10.171)	474(15.653)	294(11.675)	296(8.658)	175(5.760)	
Mexican	949(7.959)	256(7.991)	214(8.961)	286(9.092)	193(5.948)	
Other race	991(10.593)	256(10.277)	218(10.728)	282(10.921)	235(10.418)	
Education						**< 0.0001**
<high school	1,675(17.775)	654(27.424)	409(20.381)	391(15.908)	221(9.188)	
High school	1,456(23.618)	482(28.894)	321(26.479)	396(23.520)	257(16.830)	
> high school	3,137(58.607)	624(43.682)	652(53.140)	953(60.572)	908(73.982)	
PIR						**< 0.0001**
< = 1.3	1924(21.401)	727(31.909)	409(21.169)	488(18.769)	300(15.051)	
1.3–3.5	2,375(34.471)	673(36.084)	588(41.383)	631(33.894)	483(28.344)	
>3.5	1969(44.128)	360(32.008)	385(37.448)	621(47.337)	603(56.605)	
UIC(ug/L)						0.752
<100	1979(32.998)	538(33.818)	447(32.027)	539(31.220)	455(34.917)	
100–299	3,086(48.779)	873(48.090)	676(49.427)	857(49.619)	680(47.996)	
> = 300	1,203(18.223)	349(18.093)	259(18.546)	344(19.161)	251(17.087)	
HT						0.073
Yes	734(12.445)	184(10.503)	164(11.365)	206(12.746)	180(14.681)	
No	5,534(87.555)	1,576(89.497)	1,218(88.635)	1,534(87.254)	1,206(85.319)	
AIT						0.051
Yes	900(15.522)	225(12.968)	208(16.212)	248(14.758)	219(18.084)	
No	5,368(84.478)	1,535(87.032)	1,174(83.788)	1,492(85.242)	1,167(81.916)	
Hyperthyroidism						0.081
Yes	19(0.178)	7(0.347)	2(0.059)	6(0.180)	4(0.117)	
No	6,249(99.822)	1753(99.653)	1,380(99.941)	1734(99.820)	1,382(99.883)	
SCHyper						**< 0.0001**
Yes	92(1.322)	35(2.875)	17(0.648)	20(0.712)	20(1.111)	
No	6,176(98.678)	1725(97.125)	1,365(99.352)	1720(99.288)	1,366(98.889)	
SCH						0.628
Yes	152(2.765)	125(8.263)	107(8.034)	142(8.905)	95(7.204)	
No	6,116(97.235)	1,635(91.737)	1,275(91.966)	1,598(91.095)	1,291(92.796)	
Hypothyroidism						0.628
Yes	469(8.116)	125(8.263)	107(8.034)	142(8.905)	95(7.204)	
No	5,799(91.884)	1,635(91.737)	1,275(91.966)	1,598(91.095)	1,291(92.796)	
Hypertension						**< 0.0001**
Yes	2,652(35.070)	866(39.845)	622(37.496)	711(37.042)	453(26.846)	
No	3,616(64.930)	894(60.155)	760(62.504)	1,029(62.958)	933(73.154)	
Diabetes						**< 0.001**
Yes	1,179(13.305)	415(15.644)	288(15.594)	311(14.008)	165(8.710)	
No	5,089(86.695)	1,345(84.356)	1,094(84.406)	1,429(85.992)	1,221(91.290)	
CVD						**< 0.0001**
Yes	712(7.952)	289(12.080)	171(9.234)	166(7.219)	86(4.078)	
No	5,556(92.048)	1,471(87.920)	1,211(90.766)	1,574(92.781)	1,300(95.922)	

a Actual frequencies (weighted percentages).

OBS, oxidative balance score; PIR, poverty to income ratio; UIC, urinary iodine concentration; HT, Hashimoto’s thyroiditis; AIT, autoimmune thyroiditis; SCHper, subclinical hyperthyroidism; SCH, subclinical hypothyroidism; CVD, cardiovascular disease.

Bold values (*P* < 0.05) indicate statistically significant associations.

### 3.2 Association between OBS and SCHyper

The association between OBS and SCHyper was illustrated in Table 2. In all regression models, the influence of OBS on SCHyper was statistically significant. In model 3, with each 1 unit increase in total OBS, the prevalence of SCHyper decreased by a factor of 7.4%[OR = 0.926, 95% CI = (0.884, 0.971), *p* = 0.002], revealing a negative correlation between OBS and the risk of SCHyper. Furthermore, when compared to the lowest quartile of total OBS, the second, third, and highest quartiles also exhibited a negative correlation with the risk of SCHyper [Q2: OR = 0.216, 95% CI = (0.100, 0.464), *p* < 0.001; Q3: OR = 0.241, 95% CI = (0.105, 0.552), *p* = 0.001; Q4: OR = 0.377, 95% CI = (0.158, 0.902), *p =* 0.030; respectively]. The findings indicated that individuals with the highest OBS exhibited a 62.3% reduced risk of developing SCHyper in comparison to those with the lowest OBS. And the negative trend remained statistically significant (*P* for trend = 0.022).

**Table 2:** The association between OBS and SCHyper.

Variables	Model 1		Model 2		Model 3	
	OR (95%CI)	*P*	OR (95%CI)	*P*	OR(95%CI)	*P*
OBS	0.928(0.889,0.968)	**<0.001**	0.926(0.882, 0.972)	**0.003**	0.926(0.884, 0.971)	**0.002**
Categories						
Q1	Ref		Ref		Ref	
Q2	0.220(0.107,0.455)	**<0.001**	0.218(0.101, 0.467)	**<0.001**	0.216(0.100, 0.464)	**<0.001**
Q3	0.242(0.120,0.490)	**<0.001**	0.240(0.105, 0.547)	**0.001**	0.241(0.105, 0.552)	**0.001**
Q4	0.380(0.200,0.720)	**0.004**	0.382(0.161, 0.906)	**0.030**	0.377(0.158, 0.902)	**0.030**
p for trend		**0.004**		**0.023**		**0.022**

Model 1 was not adjusted for any covariates. Model 2 incorporated adjustments for various factors, including gender, race, education level, age, UIC, and PIR. Model 3, the fully adjusted model, was adjusted for the variables in Model 2 and further adjusted to account for additional potential confounders, such as diabetes, hypertension, and CVD. OBS, oxidative balance score; SCHyper, subclinical hyperthyroidism; OR, odds ratio; CI, confidence interval; ref, reference; PIR, poverty to income ratio; UIC, urinary iodine concentration; CVD, cardiovascular disease.

Bold values (*P* < 0.05) indicate statistically significant associations.

### 3.3 Association between dietary/lifestyle OBS and SCHyper

The relationship between dietary/lifestyle OBS and SCHyper was presented in Table 3. Similar to the total OBS results, dietary OBS was negatively correlated with the risk of SCHyper [OR = 0.923, 95% CI = (0.874, 0.974), *p* = 0.005]. While lifestyle OBS were expected to function as a protective measure against SCHyper, the results failed to reach statistical significance[OR = 0.895, 95% CI = (0.646, 1.238), *p* = 0.490].

**Table 3:** The relationship between dietary/lifestyle OBS and SCHyper.

Variables	Model 1		Model 2		Model 3	
	OR (95%CI)	*P*	OR (95%CI)	*P*	OR (95%CI)	*P*
OBS.dietary	0.920(0.879,0.964)	**<0.001**	0.923(0.874, 0.974)	**0.005**	0.923(0.874, 0.974)	**0.005**
Categories						
Q1	Ref		Ref		Ref	
Q2	0.144(0.062,0.333)	**<0.0001**	0.144(0.060, 0.348)	**<0.0001**	0.143(0.059, 0.347)	**<0.001**
Q3	0.211(0.108,0.409)	**<0.0001**	0.213(0.096, 0.475)	**<0.001**	0.214(0.094, 0.485)	**<0.001**
Q4	0.403(0.212,0.766)	**0.007**	0.431(0.190, 0.979)	**0.045**	0.430(0.190, 0.970)	**0.043**
p for trend		**0.007**		**0.031**		**0.030**
OBS.lifestyle	0.936(0.701,1.250)	0.648	0.893(0.640, 1.247)	0.496	0.895(0.646, 1.238)	0.490
Categories						
Q1	Ref		Ref		Ref	
Q2	0.679(0.353,1.307)	0.240	0.622(0.311, 1.247)	0.174	0.618(0.308, 1.238)	0.167
Q3	0.893(0.461,1.731)	0.733	0.850(0.348, 2.075)	0.713	0.846(0.347, 2.059)	0.703
Q4	0.944(0.307,2.902)	0.919	0.833(0.241, 2.880)	0.766	0.830(0.243, 2.833)	0.759
p for trend		0.923		0.819		0.811

Model 1 was not adjusted for any covariates. Model 2 incorporated adjustments for various factors, including gender, race, education level, age, UIC, and PIR. Model 3, the fully adjusted model, was adjusted for the variables in Model 2 and further adjusted to account for additional potential confounders, such as diabetes, hypertension, and CVD. OBS, oxidative balance score; SCHyper, subclinical hyperthyroidism; OR, odds ratio; CI, confidence interval; ref, reference; PIR, poverty to income ratio; UIC, urinary iodine concentration; CVD, cardiovascular disease.

Bold values (*P* < 0.05) indicate statistically significant associations.

### 3.4 RCS analysis

**Figure 2:**
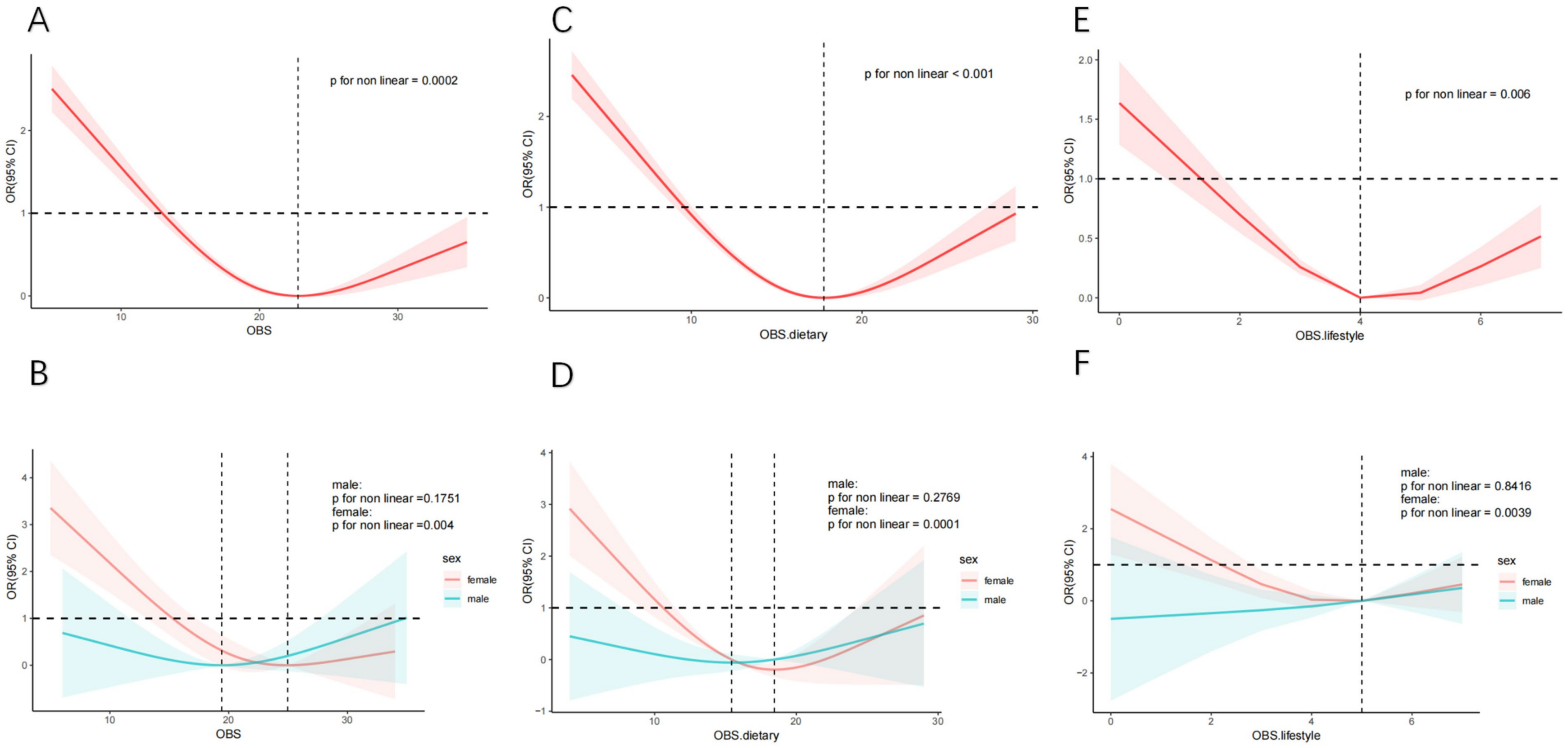
The non-linear relationships between SCHyper and OBS, dietary OBS and lifestyle OBS. Models adjust for gender, race, education level, age, UIC, PIR, diabetes, hypertension, and CVD. **(A)** OBS and SCHyper in adults; **(B)** OBS and SCHyper by sex; **(C)** dietary OBS and SCHyper in adults; **(D)** dietary OBS and SCHyper by sex; **(E)** lifestyle OBS and SCHyper in adults; **(F)** lifestyle OBS and SCHyper by sex. Abbreviations: OBS, oxidative balance score; SCHyper, subclinical hyperthyroidis; OR, odds ratio; CI, confidence interval; ref., reference; PIR, poverty to income ratio; UIC, urinary iodine concentration; CVD, cardiovascular disease.

The RCS curves were employed to assess both linear and potential non-linear relationships between OBS and SCHyper. Initially, we observed a U-shaped curve association between OBS and SCHyper (*P* for nonlinear = 0.0002) (Figure 2A). This strong U-shaped association, as depicted in Figure 2A, demonstrated a decrease in the odds ratio for SCHyper with increasing OBS levels, reaching a minimum around OBS level 22.789 before ascending once more. Additionally, the inflection point for total OBS levels in females occurred at 24.965 (*P* for nonlinear = 0.004) (Figure 2B). Consistent with the relationship, the RCS curve also revealed a U-shaped correlation between dietary/lifestyle OBS and SCHyper in all individuals, particularly in females (Figures 2C–F).

### 3.5 Subgroup analysis

The subgroup analysis of selected covariates, using multivariate logistic regression and interaction tests, was aimed to assess the heterogeneity of the relationship between OBS and SCHyper risk.

**Figure 3:**
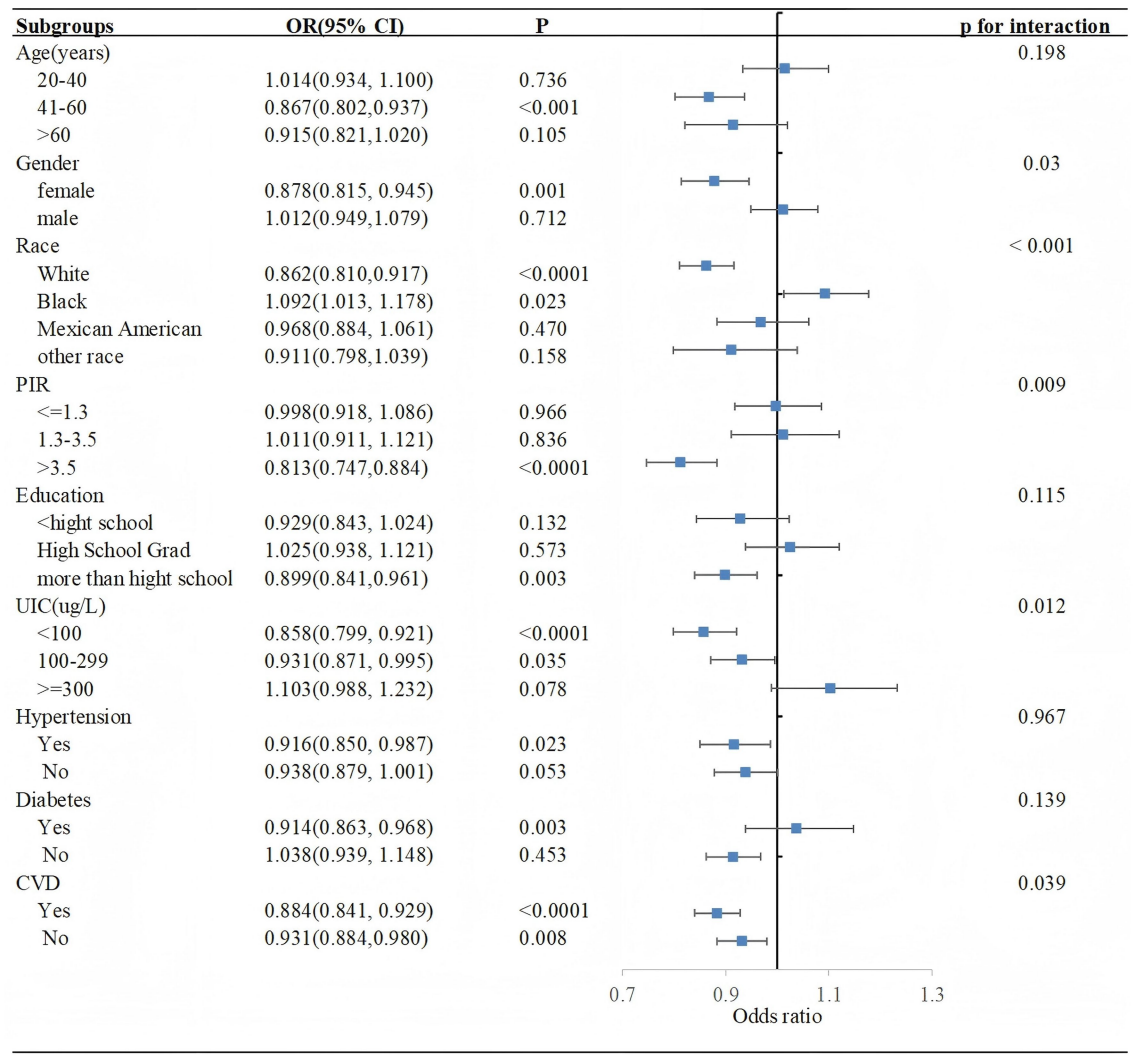
Forest plot illustrating the relationship between OBS and SCHyper risk within each subgroup. Data are presented as OR (95% CI). Each subgroup adjusted for all factors (gender, race, education level, age, UIC, PIR, diabetes, hypertension, and CVD) except the stratification factor itself. Abbreviations: OBS, oxidative balance score; PIR, poverty to income ratio; UIC, urinary iodine concentration; CVD, cardiovascular disease, CI, confidence interval; Ref, reference.

In Figure 3, we observed significant interactions in gender (*P* for interaction = 0.03), race (*P* for interaction < 0.001), PIR (*P* for interaction = 0.009) and UIC subgroup (*P* for interaction = 0.012). More precisely, the protective impact of OBS against SCHyper was more marked in the female subgroup[OR = 0.878, 95% CI = (0.815, 0.945), *p* = 0.001], the non-Hispanic White subgroup [OR = 0.862, 95% CI = (0.810, 0.917), *p* < 0.0001], the subgroup with a PIR greater than 3.5 [OR = 0.813, 95% CI = (0.747, 0.884), *p* < 0.0001], the UIC levels below 100 [OR = 0.858, 95% CI = (0.799, 0.921), *p* < 0.0001] and within the range of 100–299 [OR = 0.931, 95% CI = (0.871, 0.995), *p* = 0.035].

Furthermore, OBS was used as a categorical variable (Table 4). Compared to participants with the lowest OBS, those with the highest OBS exhibited a lower risk of SCHyper, particularly within the female subgroup [OR = 0.156, 95% CI = (0.041, 0.592), *p* = 0.005, *P* for interaction = 0.037], the non-Hispanic White subgroup [OR = 0.124, 95% CI = (0.043, 0.355), *p* < 0.001, *P* for interaction < 0.0001], the subgroup with a PIR greater than 3.5 [OR = 0.015, 95% CI = (0.002, 0.097), *p* < 0.0001, *P* for interaction < 0.001], and the subgroup with education levels more than high school [OR = 0.197, 95% CI = (0.074, 0.527), *p* = 0.006, *P* for interaction = 0.036].

**Table 4:** Relationship between OBS and SCHyper in each subgroup.

Characteristics	OBS quartiles					
	Q1	Q2	Q3	Q4	p for trend	p for interaction
Age (years)						0.079
20–40	Ref	0.774(0.213, 2.805)	0.988(0.263, 3.719)	1.481(0.375, 5.854)	0.529	
41–60	Ref	0.156(0.052,0.471)	0.072(0.016,0.323)	0.097(0.026,0.361)	**0.001**	
>60	Ref	0.067(0.013,0.342)	0.240(0.056,1.027)	0.516(0.127,2.102)	0.325	
Gender						**0.037**
Female	Ref	0.137(0.052, 0.361)	0.122(0.036, 0.416)	0.156(0.041, 0.592)	**0.005**	
Male	Ref	0.575(0.192,1.720)	0.840(0.325,2.170)	1.510(0.514,4.437)	0.428	
Race						**< 0.0001**
White	Ref	0.060(0.014,0.248)	0.082(0.024,0.284)	0.124(0.043,0.355)	**<0.001**	
Black	Ref	1.858(0.624, 5.527)	1.197(0.354, 4.052)	6.514(2.159,19.652)	**0.019**	
Mexican	Ref	0.969(0.240, 3.907)	0.609(0.175, 2.115)	1.112(0.288, 4.296)	0.917	
Other race	Ref	0.029(0.003, 0.285)	0.704(0.158, 3.131)	0.102(0.009, 1.105)	0.188	
PIR						**< 0.001**
< = 1.3	Ref	0.501(0.131, 1.918)	0.368(0.115, 1.185)	1.807(0.514, 6.354)	0.629	
1.3–3.5	Ref	0.449(0.077, 2.624)	0.613(0.139, 2.700)	1.635(0.497, 5.379)	0.512	
> 3.5	Ref	0.064(0.015,0.271)	0.091(0.026,0.314)	0.015(0.002,0.097)	**<0.0001**	
Education						**0.036**
<high school	Ref	0.303(0.054, 1.712)	0.210(0.095, 0.467)	1.054(0.364, 3.055)	0.369	
High School	Ref	0.814(0.183, 3.630)	2.284(0.755, 6.912)	1.275(0.094,17.271)	0.472	
> high school	Ref	0.111(0.036,0.337)	0.109(0.030,0.393)	0.197(0.074,0.527)	0.006	
UIC(ug/L)						0.067
<100	Ref	0.058(0.013, 0.269)	0.083(0.015, 0.455)	0.087(0.032, 0.234)	<0.001	
100–299	Ref	0.259(0.084, 0.801)	0.345(0.143, 0.828)	0.486(0.138, 1.712)	0.188	
> = 300	Ref	1.923(0.316,11.713)	2.071(0.245,17.532)	7.132(1.130,45.025)	0.056	
Hypertension						0.79
Yes	Ref	0.326(0.093, 1.140)	0.339(0.094, 1.228)	0.326(0.082, 1.302)	0.097	
No	Ref	0.183(0.061, 0.549)	0.220(0.073, 0.665)	0.429(0.142, 1.295)	0.114	
Diabetes						0.147
Yes	Ref	1.173(0.229, 6.019)	0.706(0.190, 2.630)	2.252(0.436,11.619)	0.547	
No	Ref	0.164(0.070, 0.385)	0.223(0.095, 0.524)	0.304(0.107, 0.859)	0.022	
CVD						0.243
Yes	Ref	0.087(0.007, 1.108)	0.012(0.000, 0.556)	1.432(0.317, 6.456)	0.059	
No	Ref	0.230(0.102,0.516)	0.266(0.108,0.653)	0.363(0.142,0.924)	0.037	

Data are presented as OR (95% CI). Each subgroup adjusted for all factors (gender, race, education level, age, UIC, PIR, diabetes, hypertension, and CVD) except the stratification factor itself. OBS, oxidative balance score; PIR, poverty to income ratio; UIC, urinary iodine concentration; CVD, cardiovascular disease, CI, confidence interval; Ref, reference.

Bold values (*P* < 0.05) indicate statistically significant associations.

## 4 Discussion

Our study is a cross-sectional investigation into the association between the OBS and the prevalence of thyroid dysfunction among adults in the United States. The research indicated that OBS was inversely correlated with the prevalence of SCHyper, suggesting that higher OBS scores are associated with a decreased risk of SCHyper. Furthermore, it was observed that dietary OBS also had an inverse relationship with the prevalence of SCHyper, whereas the correlation between lifestyle OBS and SCHyper did not reach statistical significance. A subgroup analysis was conducted, revealing that the protective effect of OBS against SCHyper was particularly strong in women, non-Hispanic White people, individuals with PIR greater than 3.5, and those with a UIC below 300 ug/L. Additionally, our investigation revealed a nonlinear U-shaped relationship between OBS and SCHyper.

Previous research has shown that OS is linked to both hypothyroidism and hyperthyroidism (12). The production of OS in hyperthyroidism is due to increased ROS generation, whereas in hypothyroidism, it is due to lower utilization of antioxidants. ROS contribute to damage in the thyroid and peripheral tissues. Mitochondria serve as the primary sites for the production of ROS and are also the primary targets of these potentially harmful molecules, which can induce mitochondrial dysfunction and consequently lead to metabolic disorders. A mini-review confirmed that vitamin E supplementation in experimental and functional hyperthyroidism has the potential to prevent or reduce mitochondrial oxidative dysfunction by reaching the mitochondrial membrane through different pathways (32). The finding of Gallo et al. (33) showed that patients newly diagnosed with hyperthyroidism and presenting with low levels of Se and vitamin D(Vd), supplementation with Se and Vd facilitates early control of hyperthyroidism during methimazole treatment. The reason is that low Se levels could worsen OS in hyperthyroidism by impairing the antioxidant system’s ability to counteract reactive oxygen species. When Se binds with selenoproteins, it can enhance the defense of thyroid cells against ROS (34). A randomized clinical trial (35) revealed that the OS level was markedly elevated and the total antioxidant capacity was notably lower in hyperthyroid patients treated solely with anti-thyroid drugs, compared to those who received a combination of anti-thyroid drug and antioxidant supplements within the study groups. Adjuvant treatment with antioxidants can lead to improved outcomes when combined with anti-thyroid drug therapy. Additionally, many trace elements, including Se (36), copper (37), zinc (38), and iron (39), are involved in the generation of ROS and play a crucial role in sustaining of oxidant-antioxidant balance. A recent study further supports this approach, demonstrating that targeted adjuvant therapies, such as antioxidant supplementation, effectively reduce oxidative damage and improve the prognosis of HT (40). However, disruptions in the levels of these trace elements can also result in thyroid dysfunction (41–43).

Research has shown a significant link between various lifestyle factors and thyroid dysfunction. Specifically, smoking has been identified as having an adverse impact on thyroid function, potentially triggering or exacerbating hyperthyroidism, thereby elevating the risk of its onset (44, 45). Huang et al. (46) observed that the consumption of alcohol was correlated with a reduced incidence of hypothyroidism, hyperthyroidism, and the presence of positive TPOAb. Furthermore, after accounting for confounding factors, obesity was found to be linked with a higher likelihood of overt hypothyroidism (47). And a population-based cohort study demonstrated that neither cross-sectional nor longitudinal analyses revealed any association between thyroid hormone levels and physical activity (48). However, evidence also indicates that physical activity can influence OS levels and may be linked to thyroid dysfunction (49, 50). In contrast, compared to a single marker, OBS as an indicator can assess the oxidative stress status more comprehensively (15).

In the subgroup analysis and interaction terms of this study, it was found that the protective effect of OBS against SCHyper was particularly strong in women. These could be attributed to several factors. First, women may have a stronger antioxidant activity in their bodies (51), which helps combat OS caused by elevated thyroid hormone levels. Furthermore, estrogen can induce a decrease in the expression and activity of NADPH oxidase in females (52), while also enhancing the activity of glutathione peroxidase and superoxide dismutase (52–54). These enzymatic changes further enhance the protective benefits of OBS for women. However, the exact mechanisms may involve multiple factors and require more research to clarify.

The study by Díez et al. (55) indicates that socioeconomic conditions are associated with the prevalence of thyroid disorders. Specifically, the incidences of both hypothyroidism and hyperthyroidism exhibit an upward trend with decreasing income levels. Hiza et al. (56) proposed in their study that the level of education acts as an indicator of an individual’s ability to apply nutritional knowledge to healthier dietary practices. Individuals who have attained a university degree generally exhibit higher quality diets than those with lower levels of education. Those with higher education tend to have a more elevated economic status and are typically more attentive to their dietary habits and overall lifestyle. Furthermore, prior research has indicated that Black race, females, and older individuals were risk factors for hyperthyroidism (57). Educational and income disparities contribute to variations in nutritional intake across different racial groups. This dietary difference between races may be one of the main factors contributing to the variation in rates of thyroid dysfunction. Globally, iodine deficiency, aside from the administration of levothyroxine, is the primary risk factor for subclinical hyperthyroidism (58). Iodine functions as an antioxidant, inhibiting the formation of free radicals and ROS. Iodine deficiency can lead to an elevated incidence of thyroid nodules, which is subsequently associated with an increase in hyperthyroidism cases (59–61). Our study also indicates that for non-Hispanic White people, individuals with PIR greater than 3.5, and those with a UIC below 300 ug/L, the protective effect of OBS against SCHyper was particularly strong. These findings suggest that nutritional intervention measures may need to be tailored on an individual basis according to gender, race, economic status, and specific biomarker levels to achieve optimal health outcomes.

Although our research has achieved certain results, it must also be acknowledged that there are some limitations. Firstly, since the data comes from the NHANES database, which employs a cross-sectional design, it is not possible to determine the causal relationship between OBS and SCHyper. Secondly, the assessment of OBS is based on self-reported data and dietary recalls, which could introduce recall bias. Thirdly, individuals with higher OBS may engage in unmeasured health-promoting behaviors, which could independently reduce the risk of SCHyper. Lastly, despite our utmost efforts to eliminate potential confounders, there are still some factors that cannot be entirely controlled and could influence the final outcome.

## 5 Conclusion

In summary, our study indicated that there is a negative correlation between OBS and the risk of SCHyper. And the protective effect of OBS against SCHyper was particularly strong in women, non-Hispanic White people, individuals with PIR greater than 3.5, and those with a UIC below 300 ug/L. Furthermore, our investigation revealed a nonlinear U-shaped relationship between OBS and SCHyper. The findings suggest that an increased intake of antioxidants and reduced exposure to pro-oxidants may help lower the risk of SCHyper. Future longitudinal studies can be conducted to further validate the causal relationship between OBS and SCHyper and their potential pathological mechanisms.

## Data Availability

All the data points are available at the website (https://www.cdc.gov/nchs/nhanes/index.htm).

## References

[1] Chaker L Bianco AC Jonklaas J Peeters RP. Hypothyroidism. Lancet. (2017) 390:1550–62. doi: 10.1016/s0140-6736(17)30703-1, PMID: 28336049 PMC6619426

[2] Ross DS Burch HB Cooper DS Greenlee MC Laurberg P Maia AL. 2016 American Thyroid Association guidelines for diagnosis and Management of Hyperthyroidism and Other Causes of thyrotoxicosis. Thyroid. (2016) 26:1343–421. doi: 10.1089/thy.2016.0229, PMID: 27521067

[3] Antonelli A Ferrari SM Corrado A Di Domenicantonio A Fallahi P. Autoimmune thyroid disorders. Autoimmun Rev. (2015) 14:174–80. doi: 10.1016/j.autrev.2014.10.016 25461470

[4] Chaker L Korevaar TI Medici M Uitterlinden AG Hofman A Dehghan A. Thyroid function characteristics and determinants: the Rotterdam study. Thyroid. (2016) 26:1195–204. doi: 10.1089/thy.2016.0133, PMID: 27484151

[5] Fu J Zhang G Xu P Guo R Li J Guan H. Seasonal changes of thyroid function parameters in women of reproductive age between 2012 and 2018: a retrospective, observational, single-center study. Front Endocrinol. (2021) 12:719225. doi: 10.3389/fendo.2021.719225, PMID: 34539571 PMC8443767

[6] Yeo Y Han K Shin DW Kim D Jeong SM Chun S. Changes in smoking, alcohol consumption, and the risk of thyroid Cancer: a population-based Korean cohort study. Cancers. (2021) 13:343. doi: 10.3390/cancers13102343, PMID: 34066228 PMC8150527

[7] Bellastella G Scappaticcio L Caiazzo F Tomasuolo M Carotenuto R Caputo M. Mediterranean diet and thyroid: an interesting Alliance. Nutrients. (2022) 14:14. doi: 10.3390/nu14194130, PMID: 36235782 PMC9571437

[8] Liu S Lu C He L Shan Z Teng W Li Y. Vitamin E intake and prevalence rates of thyroid dysfunction and autoimmune thyroiditis: a cross-sectional analysis of NHANES data. Thyroid. (2024) 34:753–63. doi: 10.1089/thy.2023.0561, PMID: 38534308

[9] Zhou Q Xue S Zhang L Chen G. Trace elements and the thyroid. Front Endocrinol. (2022) 13:904889. doi: 10.3389/fendo.2022.904889, PMID: 36353227 PMC9637662

[10] Wróblewski M Wróblewska J Nuszkiewicz J Pawłowska M Wesołowski R Woźniak A. The role of selected trace elements in Oxidoreductive homeostasis in patients with thyroid diseases. Int J Mol Sci. (2023) 24:24. doi: 10.3390/ijms24054840, PMID: 36902266 PMC10003705

[11] Wu J Jia C Wang Q Li X. Association between vitamin C intake and thyroid function among U.S. adults: a population-based study. Front Endocrinol. (2024) 15:1462251. doi: 10.3389/fendo.2024.1462251, PMID: 39574958 PMC11578698

[12] Resch U Helsel G Tatzber F Sinzinger H. Antioxidant status in thyroid dysfunction. Clin Chem Lab Med. (2002) 40:1132–4. doi: 10.1515/cclm.2002.198, PMID: 12521231

[13] Mancini A Di Segni C Raimondo S Olivieri G Silvestrini A Meucci E. Thyroid hormones, oxidative stress, and inflammation. Mediat Inflamm. (2016) 2016:6757154–12. doi: 10.1155/2016/6757154, PMID: 27051079 PMC4802023

[14] Zhang W Peng SF Chen L Chen HM Cheng XE Tang YH. Association between the oxidative balance score and telomere length from the National Health and nutrition examination survey 1999-2002. Oxidative Med Cell Longev. (2022) 2022:1345071–11. doi: 10.1155/2022/1345071, PMID: 35186180 PMC8850082

[15] Talavera-Rodriguez I Fernandez-Lazaro CI Hernández-Ruiz Á Hershey MS Galarregui C Sotos-Prieto M. Association between an oxidative balance score and mortality: a prospective analysis in the SUN cohort. Eur J Nutr. (2023) 62:1667–80. doi: 10.1007/s00394-023-03099-8, PMID: 36781422 PMC10195723

[16] Chen K Li S Xie Z Liu Y Li Y Mai J. Association between oxidative balance score, systemic inflammatory response index, and cardiovascular disease risk: a cross-sectional analysis based on NHANES 2007-2018 data. Front Nutr. (2024) 11:1374992. doi: 10.3389/fnut.2024.1374992, PMID: 38899319 PMC11186475

[17] Xu Z Liu D Zhai Y Tang Y Jiang L Li L. Association between the oxidative balance score and all-cause and cardiovascular mortality in patients with diabetes and prediabetes. Redox Biol. (2024) 76:103327. doi: 10.1016/j.redox.2024.103327, PMID: 39186882 PMC11389538

[18] Sohouli MH Baniasadi M Hernández-Ruiz Á Melekoglu E Zendehdel M José Soto-Méndez M. Adherence to oxidative balance scores is associated with a reduced risk of breast Cancer; a case-control study. Nutr Cancer. (2023) 75:164–73. doi: 10.1080/01635581.2022.2102658, PMID: 35875876

[19] Yang X Sun H Zhang W Hou S Lin J Chen Z. Association of oxidative balance score with epilepsy and moderate to severe depression: insights from the NHANES study. J Affect Disord. (2024) 363:292–9. doi: 10.1016/j.jad.2024.07.115, PMID: 39029686

[20] Chen Q Bao W Kong X Zhu J Hou S Zhang Y. Association between the oxidative balance score and kidney stones in adults. World J Urol. (2024) 42:425. doi: 10.1007/s00345-024-05144-5 39037613

[21] Su Z Ding P Su W Li X Li Y Li X. Association between oxidative balance score and female infertility from the national health and nutrition examination survey 2013-2018. Front Endocrinol. (2024) 15:1386021. doi: 10.3389/fendo.2024.1386021, PMID: 39140031 PMC11319134

[22] Song L Zhou H Yang Q He N Fu F Li W. Association between the oxidative balance score and thyroid function: results from the NHANES 2007-2012 and Mendelian randomization study. PLoS One. (2024) 19:e0298860. doi: 10.1371/journal.pone.0298860, PMID: 38498431 PMC10947682

[23] Liu X Liu X Wang Y Zeng B Zhu B Dai F. Association between depression and oxidative balance score: National Health and nutrition examination survey (NHANES) 2005-2018. J Affect Disord. (2023) 337:57–65. doi: 10.1016/j.jad.2023.05.071, PMID: 37244542

[24] Garber JR Cobin RH Gharib H Hennessey JV Klein I Mechanick JI. Clinical practice guidelines for hypothyroidism in adults: cosponsored by the American Association of Clinical Endocrinologists and the American Thyroid Association. Endocr Pract. (2012) 18:988–1028. doi: 10.4158/ep12280.Gl 23246686

[25] Airaksinen J Komulainen K García-Velázquez R Määttänen I Gluschkoff K Savelieva K. Subclinical hypothyroidism and symptoms of depression: evidence from the National Health and nutrition examination surveys (NHANES). Compr Psychiatry. (2021) 109:152253. doi: 10.1016/j.comppsych.2021.152253, PMID: 34147730

[26] Neves JS Leitão L Baeta Baptista R Bigotte Vieira M Magriço R Viegas Dias C. Lower free triiodothyronine levels within the reference range are associated with higher cardiovascular mortality: an analysis of the NHANES. Int J Cardiol. (2019) 285:115–20. doi: 10.1016/j.ijcard.2019.03.009, PMID: 30879936

[27] Li L Ying YX Liang J Geng HF Zhang QY Zhang CR. Urinary iodine and genetic predisposition to Hashimoto's thyroiditis in a Chinese Han population: a case-control study. Thyroid. (2020) 30:1820–30. doi: 10.1089/thy.2020.0094, PMID: 32746755

[28] Yehuda M Wang CH Pak Y Chiu KC Gianoukakis AG. Parity and risk of thyroid autoimmunity based on the NHANES (2001-2002, 2007-2008, 2009-2010, and 2011-2012). J Clin Endocrinol Metab. (2017) 102:3437–42. doi: 10.1210/jc.2017-00290, PMID: 28911140

[29] Lei X Xu Z Chen W. Association of oxidative balance score with sleep quality: NHANES 2007-2014. J Affect Disord. (2023) 339:435–42. doi: 10.1016/j.jad.2023.07.040 37442450

[30] Tian X Xue B Wang B Lei R Shan X Niu J. Physical activity reduces the role of blood cadmium on depression: a cross-sectional analysis with NHANES data. Environ Pollut. (2022) 304:119211. doi: 10.1016/j.envpol.2022.119211 35341822

[31] World Health O. Assessment of iodine deficiency disorders and monitoring their elimination: a guide for programme managers. 3rd ed. Geneva: World Health Organization (2007).

[32] Napolitano G Fasciolo G Di Meo S Venditti P. Vitamin E supplementation and mitochondria in experimental and functional hyperthyroidism: a Mini-review. Nutrients. (2019) 11:11. doi: 10.3390/nu11122900, PMID: 31805673 PMC6950234

[33] Gallo D Mortara L Veronesi G Cattaneo SA Genoni A Gallazzi M. Add-on effect of selenium and vitamin D combined supplementation in early control of Graves' disease hyperthyroidism during Methimazole treatment. Front Endocrinol. (2022) 13:886451. doi: 10.3389/fendo.2022.886451, PMID: 35784564 PMC9240752

[34] Winther KH Rayman MP Bonnema SJ Hegedüs L. Selenium in thyroid disorders - essential knowledge for clinicians. Nat Rev Endocrinol. (2020) 16:165–76. doi: 10.1038/s41574-019-0311-6, PMID: 32001830

[35] Sultana DR Shahin AD Md Jawadul H. Measurement of oxidative stress and total antioxidant capacity in hyperthyroid patients following treatment with carbimazole and antioxidant. Heliyon. (2022) 8:e08651. doi: 10.1016/j.heliyon.2021.e08651, PMID: 35028444 PMC8741446

[36] Guillin OM Vindry C Ohlmann T Chavatte L. Selenium, Selenoproteins and viral infection. Nutrients. (2019) 11:11. doi: 10.3390/nu11092101, PMID: 31487871 PMC6769590

[37] Pham AN Xing G Miller CJ Waite TD. Fenton-like copper redox chemistry revisited: hydrogen peroxide and superoxide mediation of copper-catalyzed oxidant production. J Catal. (2013) 301:54–64. doi: 10.1016/j.jcat.2013.01.025

[38] Choi S Liu X Pan Z. Zinc deficiency and cellular oxidative stress: prognostic implications in cardiovascular diseases. Acta Pharmacol Sin. (2018) 39:1120–32. doi: 10.1038/aps.2018.25, PMID: 29926844 PMC6289396

[39] Maouche N Meskine D Alamir B Koceir EA. Trace elements profile is associated with insulin resistance syndrome and oxidative damage in thyroid disorders: manganese and selenium interest in Algerian participants with dysthyroidism. J Trace Elem Med Biol. (2015) 32:112–21. doi: 10.1016/j.jtemb.2015.07.002, PMID: 26302919

[40] da Silva GB Yamauchi MA Bagatini MD. Oxidative stress in Hashimoto's thyroiditis: possible adjuvant therapies to attenuate deleterious effects. Mol Cell Biochem. (2023) 478:949–66. doi: 10.1007/s11010-022-04564-4, PMID: 36168075

[41] Pop V Krabbe J Maret W Rayman M. Plasma mineral (selenium, zinc or copper) concentrations in the general pregnant population, adjusted for supplement intake, in relation to thyroid function. Br J Nutr. (2021) 125:71–8. doi: 10.1017/s000711452000255x, PMID: 32660679

[42] Giray B Arnaud J Sayek I Favier A Hincal F. Trace elements status in multinodular goiter. J Trace Elem Med Biol. (2010) 24:106–10. doi: 10.1016/j.jtemb.2009.11.003, PMID: 20413068

[43] Hanif S Ilyas A Shah MH. Statistical evaluation of trace metals, TSH and T(4) in blood serum of thyroid disease patients in comparison with controls. Biol Trace Elem Res. (2018) 183:58–70. doi: 10.1007/s12011-017-1137-5, PMID: 28836155

[44] Andersen SL Olsen J Wu CS Laurberg P. Smoking reduces the risk of hypothyroidism and increases the risk of hyperthyroidism: evidence from 450,842 mothers giving birth in Denmark. Clin Endocrinol. (2014) 80:307–14. doi: 10.1111/cen.12279, PMID: 23808881

[45] Hou X Li Y Li J Wang W Fan C Wang H. Development of thyroid dysfunction and autoantibodies in Graves' multiplex families: an eight-year follow-up study in Chinese Han pedigrees. Thyroid. (2011) 21:1353–8. doi: 10.1089/thy.2011.0035, PMID: 22029718

[46] Huang Y Cai L Zheng Y Pan J Li L Zong L. Association between lifestyle and thyroid dysfunction: a cross-sectional epidemiologic study in the she ethnic minority group of Fujian Province in China. BMC Endocr Disord. (2019) 19:83. doi: 10.1186/s12902-019-0414-z, PMID: 31362731 PMC6668292

[47] Mahdavi M Amouzegar A Mehran L Madreseh E Tohidi M Azizi F. Investigating the prevalence of primary thyroid dysfunction in obese and overweight individuals: Tehran thyroid study. BMC Endocr Disord. (2021) 21:89. doi: 10.1186/s12902-021-00743-4, PMID: 33931052 PMC8086289

[48] Roa Dueñas OH Koolhaas C Voortman T Franco OH Ikram MA Peeters RP. Thyroid function and physical activity: a population-based cohort study. Thyroid. (2021) 31:870–5. doi: 10.1089/thy.2020.0517, PMID: 33198599

[49] Militello R Luti S Gamberi T Pellegrino A Modesti A Modesti PA. Physical activity and oxidative stress in aging. Antioxidants. (2024) 13:13. doi: 10.3390/antiox13050557, PMID: 38790662 PMC11117672

[50] Friedenreich CM Ryder-Burbidge C McNeil J. Physical activity, obesity and sedentary behavior in cancer etiology: epidemiologic evidence and biologic mechanisms. Mol Oncol. (2021) 15:790–800. doi: 10.1002/1878-0261.12772, PMID: 32741068 PMC7931121

[51] Bhatia K Elmarakby AA El-Remessy AB Sullivan JC. Oxidative stress contributes to sex differences in angiotensin II-mediated hypertension in spontaneously hypertensive rats. Am J Physiol Regul Integr Comp Physiol. (2012) 302:R274–82. doi: 10.1152/ajpregu.00546.2011, PMID: 22049231 PMC3349386

[52] Miller AA Drummond GR Mast AE Schmidt HH Sobey CG. Effect of gender on NADPH-oxidase activity, expression, and function in the cerebral circulation: role of estrogen. Stroke. (2007) 38:2142–9. doi: 10.1161/strokeaha.106.477406, PMID: 17525399

[53] Kander MC Cui Y Liu Z. Gender difference in oxidative stress: a new look at the mechanisms for cardiovascular diseases. J Cell Mol Med. (2017) 21:1024–32. doi: 10.1111/jcmm.13038, PMID: 27957792 PMC5387169

[54] Allegra A Caserta S Genovese S Pioggia G Gangemi S. Gender differences in oxidative stress in relation to Cancer susceptibility and survival. Antioxidants. (2023) 12:12. doi: 10.3390/antiox12061255, PMID: 37371985 PMC10295142

[55] Díez JJ Iglesias P. Prevalence of thyroid dysfunction and its relationship to income level and employment status: a nationwide population-based study in Spain. Hormones (Athens). (2023) 22:243–52. doi: 10.1007/s42000-023-00435-9, PMID: 36805924

[56] Hiza HA Casavale KO Guenther PM Davis CA. Diet quality of Americans differs by age, sex, race/ethnicity, income, and education level. J Acad Nutr Diet. (2013) 113:297–306. doi: 10.1016/j.jand.2012.08.011, PMID: 23168270

[57] Zhang X Wang Y Wang H Zhang X. Trends in prevalence of thyroid dysfunction and its associations with mortality among US participants, 1988-2012. J Clin Endocrinol Metab. (2024) 109:e657–66. doi: 10.1210/clinem/dgad558 37738422

[58] Taylor PN Albrecht D Scholz A Gutierrez-Buey G Lazarus JH Dayan CM. Global epidemiology of hyperthyroidism and hypothyroidism. Nat Rev Endocrinol. (2018) 14:301–16. doi: 10.1038/nrendo.2018.18, PMID: 29569622

[59] Zimmermann MB Boelaert K. Iodine deficiency and thyroid disorders. Lancet Diabetes Endocrinol. (2015) 3:286–95. doi: 10.1016/s2213-8587(14)70225-6, PMID: 25591468

[60] Liu XH Chen GG Vlantis AC van Hasselt CA. Iodine mediated mechanisms and thyroid carcinoma. Crit Rev Clin Lab Sci. (2009) 46:302–18. doi: 10.3109/10408360903306384, PMID: 19958216

[61] Laurberg P Cerqueira C Ovesen L Rasmussen LB Perrild H Andersen S. Iodine intake as a determinant of thyroid disorders in populations. Best Pract Res Clin Endocrinol Metab. (2010) 24:13–27. doi: 10.1016/j.beem.2009.08.013, PMID: 20172467

