# Supplementary material for: Association between oxidative balance score and prevalence rates of thyroid dysfunction and autoimmune thyroiditis among U.S. adults: evidence from epidemiological studies
Source: Front Nutr. 2025 May 15;12:1592577. doi: 10.3389/fnut.2025.1592577 (PMC12119315; doi:10.3389/fnut.2025.1592577)
Supplement: Supplementary file 1 [file Table_1.DOC]

**Supplementary Table 1.** Baseline characteristics of the NHANES(2007-2012) study population in dietary OBS quartiles

| **Characreristics** | **OBS.dietary quartiles** | | | | | P-value |
| --- | --- | --- | --- | --- | --- | --- |
|  | Overall | Q1 | Q2 | Q3 | Q4 |  |
| **Age(years)** |  |  |  |  |  | **0.001** |
| 20-40 | 2132(39.227) | 543(41.028) | 462(37.167) | 641(37.000) | 486(42.104) |  |
| 41-60 | 2076(38.990) | 529(35.967) | 437(36.804) | 626(40.542) | 484(41.810) |  |
| >60 | 2060(21.784) | 642(23.005) | 522(26.029) | 567(22.458) | 329(16.086) |  |
| **Gender** |  |  |  |  |  | 0.363 |
| female | 3167(52.823) | 804(51.018) | 742(51.576) | 960(54.892) | 661(53.025) |  |
| male | 3101(47.177) | 910(48.982) | 679(48.424) | 874(45.108) | 638(46.975) |  |
| **Race** |  |  |  |  |  | **< 0.0001** |
| White | 3089(71.277) | 752(66.619) | 671(68.000) | 940(72.071) | 726(77.545) |  |
| Black | 1239(10.171) | 442(14.686) | 307(12.256) | 312( 8.518) | 178( 6.153) |  |
| Mexican | 949( 7.959) | 258(8.342) | 213(8.363) | 285(8.832) | 193(6.153) |  |
| other race | 991(10.593) | 262(10.353) | 230(11.381) | 297(10.579) | 202(10.150) |  |
| **Education** |  |  |  |  |  | **< 0.0001** |
| <high school | 1675(17.775) | 628(26.249) | 416(20.436) | 414(16.023) | 217( 9.629) |  |
| High School | 1456(23.618) | 450(28.019) | 348(26.785) | 408(22.844) | 250(17.662) |  |
| > high school | 3137(58.607) | 636(45.732) | 657(52.778) | 1012(61.133) | 832(72.709) |  |
| **PIR** |  |  |  |  |  | **< 0.0001** |
| <=1.3 | 1924(21.401) | 692(30.380) | 408(20.735) | 530(19.756) | 294(15.541) |  |
| 1.3-3.5 | 2375(34.471) | 657(36.570) | 611(41.984) | 652(33.064) | 455(27.707) |  |
| >3.5 | 1969(44.128) | 365(33.051) | 402(37.282) | 652(47.181) | 550(56.752) |  |
| **UIC(ug/L)** |  |  |  |  |  | 0.983 |
| <100 | 1979(32.998) | 531(33.993) | 452(32.208) | 597(32.501) | 399(33.364) |  |
| 100-299 | 3086(48.779) | 837(47.903) | 705(49.202) | 894(48.939) | 650(49.041) |  |
| >=300 | 1203(18.223) | 346(18.104) | 264(18.590) | 343(18.560) | 250(17.595) |  |
| **HT** |  |  |  |  |  | 0.129 |
| Yes | 734(12.445) | 178(10.589) | 174(11.827) | 215(12.658) | 167(14.474) |  |
| No | 5534(87.555) | 1536(89.411) | 1247(88.173) | 1619(87.342) | 1132(85.526) |  |
| **AIT** |  |  |  |  |  | 0.104 |
| Yes | 900(15.522) | 217(13.018) | 217(15.395) | 263(15.705) | 203(17.775) |  |
| No | 5368(84.478) | 1497(86.982) | 1204(84.605) | 1571(84.295) | 1096(82.225) |  |
| **Hyperthyroidism** |  |  |  |  |  | 0.079 |
| Yes | 19( 0.178) | 7(0.355) | 2(0.045) | 6(0.176) | 4(0.128) |  |
| No | 6249(99.822) | 1707(99.645) | 1419(99.955) | 1828(99.824) | 1295(99.872) |  |
| **SCHyper** |  |  |  |  |  | **< 0.0001** |
| Yes | 92( 1.322) | 36(3.064) | 14(0.452) | 22(0.661) | 20(1.257) |  |
| No | 6176(98.678) | 1678(96.936) | 1407(99.548) | 1812(99.339) | 1279(98.743) |  |
| **SCH** |  |  |  |  |  | 0.200 |
| Yes | 152( 2.765) | 54(3.953) | 31(2.679) | 38(1.847) | 29(2.864) |  |
| No | 6116(97.235) | 1660(96.047) | 1390(97.321) | 1796(98.153) | 1270(97.136) |  |
| **Hypothyroidism** |  |  |  |  |  | 0.681 |
| Yes | 469( 8.116) | 119(8.180) | 114(8.028) | 148(8.863) | 88(7.197) |  |
| No | 5799(91.884) | 1595(91.820) | 1307(91.972) | 1686(91.137) | 1211(92.803) |  |
| **Hypertension** |  |  |  |  |  | **0.012** |
| Yes | 2652(35.070) | 806(38.512) | 652(37.516) | 738(34.955) | 456(29.827) |  |
| No | 3616(64.930) | 908(61.488) | 769(62.484) | 1096(65.045) | 843(70.173) |  |
| **Diabetes** |  |  |  |  |  | **0.008** |
| Yes | 1179(13.305) | 372(13.775) | 301(15.249) | 334(14.445) | 172( 9.738) |  |
| No | 5089(86.695) | 1342(86.225) | 1120(84.751) | 1500(85.555) | 1127(90.262) |  |
| **CVD** |  |  |  |  |  | **< 0.0001** |
| Yes | 712( 7.952) | 269(11.219) | 186(10.176) | 166( 6.618) | 91( 4.592) |  |
| No | 5556(92.048) | 1445(88.781) | 1235(89.824) | 1668(93.382) | 1208(95.408) |  |

Notes: **^a^**Actual frequencies (weighted percentages).

Abbreviations: OBS.dietary, dietary oxidative balance score; PIR, poverty to income ratio; UIC, urinary iodine concentration; HT, Hashimoto’s thyroiditis; AIT, autoimmune thyroiditis; SCH, subclinical hypothyroidism; SCHper, Subclinical hyperthyroidism; CVD, cardiovascular disease.
