# Supplementary material for: Association between oxidative balance score and prevalence rates of thyroid dysfunction and autoimmune thyroiditis among U.S. adults: evidence from epidemiological studies
Source: Front Nutr. 2025 May 15;12:1592577. doi: 10.3389/fnut.2025.1592577 (PMC12119315; doi:10.3389/fnut.2025.1592577)
Supplement: Supplementary file 2 [file Table_2.DOC]

**Supplementary Table 2.** Baseline characteristics of the NHANES(2007-2012) study population in lifestyle OBS quartiles

| **Characreristics** | **OBS.lifestyle** | | | | | P-value |
| --- | --- | --- | --- | --- | --- | --- |
|  | Overall | Q1 | Q2 | Q3 | Q4 |  |
| **Age(years)** |  |  |  |  |  | **0.019** |
| 20-40 | 2132(39.227) | 730(35.245) | 429(36.586) | 541(43.041) | 432(44.633) |  |
| 41-60 | 2076(38.990) | 868(41.880) | 461(40.653) | 425(36.524) | 322(34.972) |  |
| >60 | 2060(21.784) | 823(22.875) | 461(22.761) | 447(20.435) | 329(20.395) |  |
| **Gender** |  |  |  |  |  | **0.031** |
| female | 3167(52.823) | 1333(55.774) | 646(50.457) | 663(49.247) | 525(54.144) |  |
| male | 3101(47.177) | 1088(44.226) | 705(49.543) | 750(50.753) | 558(45.856) |  |
| **Race** |  |  |  |  |  | **< 0.001** |
| White | 3089(71.277) | 1161(69.395) | 646(69.388) | 707(72.989) | 575(74.597) |  |
| Black | 1239(10.171) | 607(14.106) | 266(10.581) | 232( 7.707) | 134( 5.570) |  |
| Mexican | 949( 7.959) | 333(7.313) | 225(9.048) | 248(9.862) | 143(5.775) |  |
| other race | 991(10.593) | 320( 9.185) | 214(10.983) | 226( 9.443) | 231(14.059) |  |
| **Education** |  |  |  |  |  | **< 0.0001** |
| <high school | 1675(17.775) | 812(24.474) | 366(16.932) | 326(16.124) | 171( 8.554) |  |
| High School | 1456(23.618) | 636(29.146) | 330(24.301) | 305(22.928) | 185(13.801) |  |
| > high school | 3137(58.607) | 973(46.381) | 655(58.767) | 782(60.948) | 727(77.645) |  |
| **PIR** |  |  |  |  |  | **< 0.0001** |
| <=1.3 | 1924(21.401) | 922(27.796) | 430(23.003) | 352(16.452) | 220(14.061) |  |
| 1.3-3.5 | 2375(34.471) | 905(35.777) | 488(31.665) | 572(36.912) | 410(32.164) |  |
| >3.5 | 1969(44.128) | 594(36.427) | 433(45.331) | 489(46.635) | 453(53.775) |  |
| **UIC(ug/L)** |  |  |  |  |  | 0.274 |
| <100 | 1979(32.998) | 731(30.367) | 411(32.948) | 459(33.909) | 378(36.702) |  |
| 100-299 | 3086(48.779) | 1213(51.056) | 680(47.472) | 682(49.509) | 511(45.188) |  |
| >=300 | 1203(18.223) | 477(18.577) | 260(19.580) | 272(16.582) | 194(18.110) |  |
| **HT** |  |  |  |  |  | 0.262 |
| Yes | 734(12.445) | 262(11.273) | 152(11.085) | 183(14.736) | 137(13.271) |  |
| No | 5534(87.555) | 2159(88.727) | 1199(88.915) | 1230(85.264) | 946(86.729) |  |
| **AIT** |  |  |  |  |  | 0.066 |
| Yes | 900(15.522) | 325(14.290) | 178(12.905) | 221(18.138) | 176(17.370) |  |
| No | 5368(84.478) | 2096(85.710) | 1173(87.095) | 1192(81.862) | 907(82.630) |  |
| **Hyperthyroidism** |  |  |  |  |  | **0.047** |
| Yes | 19( 0.178) | 10(0.329) | 5(0.149) | 2(0.038) | 2(0.100) |  |
| No | 6249(99.822) | 2411(99.671) | 1346(99.851) | 1411(99.962) | 1081(99.900) |  |
| **SCHyper** |  |  |  |  |  | 0.801 |
| Yes | 92( 1.322) | 32(1.471) | 19(1.003) | 25(1.316) | 16(1.390) |  |
| No | 6176(98.678) | 2389(98.529) | 1332(98.997) | 1388(98.684) | 1067(98.610) |  |
| **SCH** |  |  |  |  |  | 0.483 |
| Yes | 152( 2.765) | 59(2.457) | 36(3.447) | 35(3.233) | 22(2.067) |  |
| No | 6116(97.235) | 2362(97.543) | 1315(96.553) | 1378(96.767) | 1061(97.933) |  |
| **Hypothyroidism** |  |  |  |  |  | 0.989 |
| Yes | 469( 8.116) | 190(8.102) | 101(7.889) | 101(8.203) | 77(8.272) |  |
| No | 5799(91.884) | 2231(91.898) | 1250(92.111) | 1312(91.797) | 1006(91.728) |  |
| **Hypertension** |  |  |  |  |  | **< 0.0001** |
| Yes | 2652(35.070) | 1227(43.445) | 580(35.323) | 518(30.093) | 327(25.602) |  |
| No | 3616(64.930) | 1194(56.555) | 771(64.677) | 895(69.907) | 756(74.398) |  |
| **Diabetes** |  |  |  |  |  | **< 0.0001** |
| Yes | 1179(13.305) | 635(20.040) | 248(11.912) | 204(10.311) | 92( 6.154) |  |
| No | 5089(86.695) | 1786(79.960) | 1103(88.088) | 1209(89.689) | 991(93.846) |  |
| **CVD** |  |  |  |  |  | **< 0.0001** |
| Yes | 712( 7.952) | 348(10.538) | 151( 8.236) | 135( 5.876) | 78( 5.445) |  |
| No | 5556(92.048) | 2073(89.462) | 1200(91.764) | 1278(94.124) | 1005(94.555) |  |

Notes: **^a^**Actual frequencies (weighted percentages).

Abbreviations: OBS.lifestyle, lifestyle oxidative balance score; PIR, poverty to income ratio; UIC, urinary iodine concentration; HT, Hashimoto’s thyroiditis; AIT, autoimmune thyroiditis; SCH, subclinical hypothyroidism; SCHper, Subclinical hyperthyroidism; CVD, cardiovascular disease.
